# Is the sharp increasing trend of multiple sclerosis incidence real in Iran?

**DOI:** 10.1186/s12883-020-02031-5

**Published:** 2021-01-07

**Authors:** Ali Hosseinzadeh, Behnaz Sedighi, Jamshid Kermanchi, Mohammad Heidari, Ali Akbar Haghdoost

**Affiliations:** 1 Department of Epidemiology, School of Public Health, Shahroud University of Medical Sciences, Shahroud, Iran; 2 Neurology Research Center, Kerman University of Medical Science, Kerman, Iran; 3 Deputy of Curative Affairs, Ministry of Health and Medical Education (MOHME, Tehran, Iran; 4 Clinical Research Development Unit of Imam Khomeini Hospital, Urmia University of Medical Sciences, Urmia, Iran; 5 HIV/STI Surveillance Research Center, and WHO Collaborating Center for HIV Surveillance, Institute for Futures Studies in Health, Kerman University of Medical Sciences, Kerman, Iran

**Keywords:** Multiple sclerosis, Autoimmune diseases, Incidence, Increasing, Joinpoint regression

## Abstract

**Background:**

Some epidemiologic studies have reported a sharp increase in multiple sclerosis (MS) incidence in different provinces in Iran. This report aimed to investigate more closely the increasing trend of MS incidence in the past 10 years in Iran.

**Methods:**

In this longitudinal study, the data for all MS patients meeting the McDonald criteria were obtained from a national registry, coordinated by the Ministry of Health (MOH). Joinpoint (JP) regression was used for time trend analysis of MS incidence and determine the optimal number of significant joinpoints. Finally, an annual percentage change (APC) in MS incidence for each segment of the trend line was estimated with 95% confidence interval.

**Results:**

The mean age of the patients and the mean annual incidence rate of MS were 30.9 ± 1.1 and 5.3 ± 1.9 per 100,000 population, respectively. The overall incidence rate of MS had increased significantly from 2.14 in 2006 to its peak (7.5) in 2014, per 100,000 population (APC = 12%, *P* < 0.001). The first JP was observed in 2011 in both male and female groups. The overall APC in the first segment was 22.6% (17.2–28.2%, *p* < 0.01). Besides, the corresponding APC values for males and females were 22.1% (14.7–30%, *p* < 0.01) and 22.5% (17.5–27.8%, *p* < 0.01), respectively. After 2011, the MS incidence underwent a more or less decreasing trend in both genders.

**Conclusion:**

Contrary to previous studies, the MS incidence trend in Iran was rising just before 2011, and in the recent decade, Iran has a stable rate of MS cases.

## Background

Multiple sclerosis (MS) is a central nervous system disease with unknown etiology which usually manifests in the third or fourth decades of life [1]. Evidence has revealed an increasing trend in the incidence and prevalence of MS in recent decades worldwide [2–4]. Likewise, several studies have reported a sharp increase in MS incidence in different provinces of Iran. However, it is unclear whether this upward trend is due to improvement in case ascertainment or increased incidence of MS. [5–8] Iran is the second-largest country in the Middle East with a large young population. The country has experienced significant industrial and technological growth in recent decades [9]. Therefore, most of the studies conducted in Iran have attributed the increasing trend in MS incidence to environmental factors and lifestyle changes as a result the rising trend of industrialization and urbanization in the country [5–8, 10, 11]. However, some factors may lead to a false increase in MS incidence including better and faster diagnosis, availability of paraclinical and MRI tests, increased number of neurologists, increased public awareness and information, improvement of the disease registration system, and changing diagnostic criteria. Previous studies have paid less attention to the above-mentioned factors, while these factors appear to play an essential role in the increasing trend of MS in Iran. Considering this gap in the literature, this study aimed to investigate more closely the increasing trend of MS incidence in the past 10 years in Iran.

## Methods

To answer the research question, we used the data published via the Iranian monitoring and treatment surveillance system for MS patients from 2006 to 2016. Because the Iranian government pays a considerable amount of treatment costs for MS patients, there is a population-based computerized registry in each province to register MS patients. Also, as patients have access to neurologists in almost all parts of Iran [12], it seems that nearly all MS patients have been registered in this registry system. In this study, the 2000–2025 WHO population was used as a standard population to remove the effect of different population age structures in different years.

For time trend analysis, we used Joinpoint software, which is appropriate for studying the changes in diseases with a low incidence rate. Unlike linear models, joinpoint regression breaks down the whole trend into different segments with positive and negative slopes of the changes. In each segment, the software fits the best line to the incidence points and annual percentage change calculated for each segment as an average rate of the change in that span of time. For this purpose, in the first step, it fits the simplest model to the data, and the analysis starts with the least number of joinpoints. Then, it checks whether more joinpoints could be fitted to the data. We chose the Monte Carlo permutation test to select the optimal number of statistically significant joinpoints. Finally, an annual percentage change (APC) in MS incidence for each segment of the trend line was estimated with 95% confidence interval. In the present study, the mean annual sex-specific prevalence rates were computed considering all patients to be alive with definite MS in the study period. We described the epidemiological characteristics of MS cases for different segments of the trends.

## Results

A total of 44,894 newly diagnosed cases (34,729 females [77.4%]) were registered in the MS registry system of MOH from March 21, 2006, to March 20, 2016. The average female-to-male ratio of the MS incidence was 3.4. The mean age of the patients was 30.9 ± 1.1 years at the time of diagnosis (30.5 ± 1.1 years in females and 31.6 ± 1.2 years in males). The mean annual MS incidence rate was 5.3 ± 1.9 per 100,000 population (8.5 ± 2.9 and 2.4 ± 0.9 in females and males, respectively).

The overall MS incidence had increased significantly from 2.2 (95% CI: 2.0–2.3) in 2006 to 6.7 (95% CI: 6.5–6.7) in 2016, per 100,000 population, with its peak 7.5 (95% CI: 7.2–7.6) in 2014 (APC = 12%, *P* < 0.001). The trend of MS incidence and APC in different age groups has been shown in Table 1.

**Table 1:** Age standardized rate of multiple sclerosis Incidence in Iran during 2006–2016 (Per100000 Persons) in different age groups in both genders

Age groups	Years Genders	2006	2007	2008	2009	2010	2011	2012	2013	2014	2015	2016	APC^a^	*P*-value
< 19	Males	0.21	0.33	0.41	0.47	0.53	0.55	0.66	0.61	0.50	0.55	0.44	7.7	0.01
	Females	1.06	1.28	1.32	1.82	1.98	2.54	2.49	2.42	2.36	1.96	1.71	5.2	0.01
	Total	0.63	0.75	0.78	1.01	1.09	1.53	1.56	1.51	1.43	1.26	1.02	6.0	0.01
20–29	Males	1.74	2.39	2.34	3.09	3.95	4.66	5.57	5.95	6.55	6.31	5.37	12.2	0.01
	Females	6.86	8.39	90.3	11.16	18.12	18.67	22.19	22.33	23.83	20.61	19.78	6.1	0.4
	Total	4.28	5.37	5.65	7.10	9.30	11.66	12.50	12.07	12.53	10.85	9.90	9.3	0.01
30–39	Males	1.79	3.14	2.68	3.14	4.05	5.62	5.85	5.51	6.66	6.49	5.93	12.0	0.01
	Females	6.83	11.20	11.78	14.66	19.54	21.22	21.25	20.79	21.77	21.56	20.85	11.3	0.01
	Total	4.26	6.75	6.61	8.06	10.68	13.31	15.11	15.47	17.17	17.16	16.58	13.9	0.01
40–49	Males	1.08	2.05	1.63	1.98	2.62	4.10	4.07	4.11	4.33	4.44	4.33	13.7	0.01
	Females	3.42	5.72	5.93	7.80	10.48	12.16	12.41	13.60	13.82	13.05	13.52	13.6	0.01
	Total	2.23	3.72	3.53	4.54	6.09	8.09	8.72	9.58	9.87	9.48	9.63	14.8	0.01
50–59	Males	0.39	0.41	0.21	0.73	0.62	1.16	1.45	1.20	1.79	1.74	2.03	22.5	0.01
	Females	0.86	1.65	1.39	2.29	3.36	3.02	2.40	3.43	3.62	3.97	4.63	14.9	0.01
	Total	0.62	0.94	0.67	1.33	1.66	2.09	2.07	2.55	3.00	3.16	3.67	19.7	0.01
> 60	Males	–	0.04	0.07	0.13	0.07	0.17	0.06	0.20	0.28	0.36	0.25	31.1	0.01
	Females	0.08	0.30	0.04	0.34	0.54	0.44	0.23	0.36	0.57	0.40	0.48	17.1	0.01
	Total	0.04	0.15	0.06	0.23	0.28	0.31	0.16	0.31	0.48	0.43	0.41	22.3	0.01

^a^Annual percent change

**Fig. 1:**
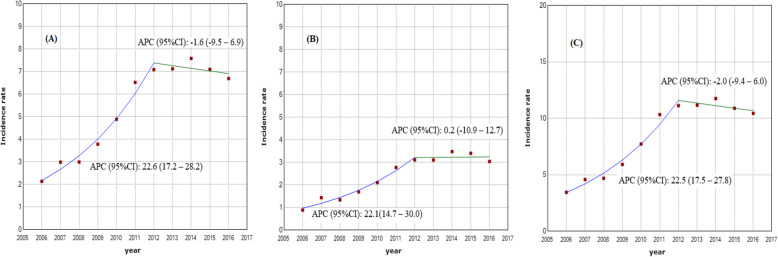
MS incidence trend over the 2006–2016 period in (**a**) Overall (**b**) Males (**c**) Females

The first joinpoint has occurred in 2011 in both genders. Figure 1 shows the APC in MS incidence before and after 2011. The overall APC in the first period (before 2011) was 22.6% (17.2–28.2%, *p* < 0.01). It was 22.1% (14.7–30%, *p* < 0.01) and 22.5% (17.5–27.8%, *p* < 0.01) in males and females, respectively. After 2011 (the second period), the trend of MS incidence was more or less decreasing. Accordingly, the APC of MS incidence was − 1.6% (− 9.5–6.9%) overall, 0.2% (− 10.9–12.7%) in males, and − 2.0% (− 9.4–6.0%) in females (Fig. 1).

Table 2 shows epidemiological features for two periods (before and after 2011) separately. The mean annual MS incidence and prevalence were 3.9 ± 1.6 and 32.7 ± 17.8 in the first period, 7.0 ± 0.3, and 62.5 ± 38.2 in the second period respectively.

**Table 2:** Epidemiological characteristics of MS in the 2006–2011 and 2012–2016 periods

	Study period					
	2006–2011			2012–2016		
	Total	Males	Females	Total	Males	Females
Mean age of patients at the diagnosis time	30.15 ± 0.62	30.79 ± 0.74	29.97 ± 0.61	31.91 ± 0.73	32.63 ± 0.72	31.69 ± 0.73
Mean new cases of MS	2861 ± 1235	635 ± 258	2226 ± 978	5546 ± 218	1271 ± 84	4275 ± 160
Mean annual MS incidence	3.90 ± 1.58	1.70 ± 0.66	6.51 ± 2.58	7.01 ± 0.27	3.18 ± 0.19	10.96 ± 0.42
Mean annual MS prevalence	32.75 ± 17.85	14.54 ± 7.48	51.46 ± 29.05	62.56 ± 38.26	28.27 ± 16.06	97.82 ± 61.58

## Discussion

Our study revealed that MS incidence increased significantly from 2.2 in 2006 to 6.7 in 2016, per 100,000 population. Our findings are in line with previous studies and show a similar trend in the same period in Iran [6, 7, 13]. According to Elhami et al. [6], the MS incidence in Tehran province has increased from 0.68/100000 in 1989 to 2.93/100000 in 2008. Furthermore, Etemadifar et al. [7] showed that the MS incidence in Isfahan province has increased from 3.64/100000 in 2006 to 9.1/100000 in 2009. Similarly, Izadi et al. [13] have reported that the MS incidence rate in Fars province had risen from 4.1/100000 in 2002 to 8.98/100000 in 2012.

In recent years, Iran has experienced significant growth in the field of medicine and access to health services. Because of such a development in the surveillance system, patients, even in deprived areas, have access to neurologists and health services through the referral system [12]. Therefore, the increase in MS incidence in Iran, especially in more developed provinces, might be the result of improved and modified diagnosis criteria (the availability of magnetic resonance imaging (MRI) and paraclinical tests), public awareness of the disease and its therapies, and improvement and the completeness of the registration system in recent years [14].

As shown in Fig. 1, the trend of MS incidence in Iran is marked by two distinct periods (i.e. the first period from 2006 to 2011 and the second period from 2012 to 2016). Although the MS incidence in the second period is high, the slope of MS incidence in the first period is higher. One of the factors that may have caused a different trend slope in the MS incidence in these two periods is the improvement of the disease registration system in the recent decade. The registration of MS patients in Iran began in 2005 [9, 15]. To the best of our knowledge, the MS registration system in Iran was not very accurate in the early years, and many patients and physicians were not aware of such a registration system. Therefore, it seems that many patients with MS had not been registered in the first years, but as time passed, the unregistered patients in the years before 2006 were gradually identified and registered in subsequent years and produced a sharp slope in the MS incidence in the first few years. The registration system has not improved in all provinces of Iran at the same time, and it developed differently across the provinces.

Also, to the best of our knowledge, the most crucial factor that may explain the increase of MS incidence rate in the second period (2012–2016) compared to the first period (2006–2011) is the change of MS diagnostic criteria in Iran in 2011. Until 2011, the McDonald’s criterion was used to diagnose MS patients In Iran [15, 16]. According to this criterion, those with 9 MS plaques in the brain are diagnosed as having MS. Changes were made to the McDonald’s criterion in 2011 and for the diagnosis of MS, 3 MS plaques were considered sufficient in the brain [16]. Therefore, this issue increased the sensitivity of this criterion for the diagnosis of MS and led to the early diagnosis of patients with MS. As a result, it could affect the MS incidence and prevalence in recent years and should be reconsidered. Another point to be considered in examining the trend of MS in Iran is the delay time from the onset of disease to the diagnosis. The mean diagnostic delay of MS was about 9 months after 2011. Therefore, the decreasing trend observed after 2011 does not seem to be due to diagnostic delay.

Another point is the precision and accuracy of the analytical methods. In this study, we used the developed software for the first time to test the changes in MS trends in Iran, which allows the data to have nonlinear changes. It is noteworthy that the joinpoint regression model is mostly used for cancer studies, although it may well be adequate for other diseases including MS. [17]

Finally, it should be pointed out that some reports have shown that the use of MRI to diagnose MS in Iran has increased considerably in recent years [14, 18]. Therefore, an increase in the incidence of MS is more likely to be due to improvement in case ascertainment than to increasing incidence, especially in more developed provinces.

## Conclusion

Contrary to previous studies, the MS trend in Iran is rising just before 2011, and in recent years, Iran has experienced a stable rate of MS cases.

## Data Availability

The datasets used during the current study are available from the corresponding author on reasonable request.

## Abbreviations

MS: Multiple sclerosis
MOH: Ministry of Health
JP: Joinpoint
APC: Annual percent change
MRI: Magnetic resonance imaging
